# Analgesic drug development: time to break the mould

**DOI:** 10.1186/1129-2377-14-S1-I2

**Published:** 2013-02-21

**Authors:** Clifford Woolf

**Affiliations:** 1 F.M. Kirby Neurobiology Center, Boston Children’s Hospital and Department of Neurobiology, Harvard Medical School, Boston MA, USA

## 

Success in the introduction of novel, effective and safe analgesics has been notably limited over the past ten years even though existing treatments have low responder rates and a high side effect burden. The pipeline of compounds acting on novel targets is very restricted and there have been many disappointments with drugs that appeared promising in preclinical studies, failing repeatedly in clinical trials. We have to recognize that something is fundamentally wrong with the industry standard approach to the selection of targets, identification of hits, validation of leads and proof of concept clinical studies. A radically alternative strategy is required, one that is driven by the patient and focused on disease phenotype. I will discuss what such an approach may look like and how it may contribute to new therapeutics.

